# Protective effect of the extremolytes ectoine and hydroxyectoine in a porcine organ culture

**DOI:** 10.1007/s00417-020-04854-x

**Published:** 2020-07-24

**Authors:** Teresa Tsai, Ana M. Mueller-Buehl, Yathavan Satgunarajah, Sandra Kuehn, H. Burkhard Dick, Stephanie C. Joachim

**Affiliations:** Experimental Eye Research Institute, University Eye Hospital, Ruhr-University Bochum, In der Schornau 23-25, 44892 Bochum, Germany

**Keywords:** Extremolytes, Ectoine, Hydroxyectoine, Hypoxia, Porcine organ culture

## Abstract

**Purpose:**

Hypoxic damage to the retina is a relevant component of neurodegenerative pathologies such as glaucoma or retinal ischemia. In porcine retina organ cultures, hypoxic damage can be induced by applying cobalt chloride (CoCl_2_). The aim of our study was to investigate possible neuroprotective effects of the extremolytes ectoine and hydroxyectoine in this hypoxia-damaged retina model.

**Methods:**

To simulate hypoxia, porcine retina organ cultures were damaged with 300 μM CoCl_2_ for 48 h starting on day 1 (*n* = 8–9/group). In order to investigate the possible neuroprotective effects of ectoine and hydroxyectoine, 0.5 mM of each extremolyte was added to the culture at the same time as the stressor and for the same duration. On day 8, the retina organ cultures were taken for (immuno)-histochemical examinations. Retinal ganglion cells (RGCs), macroglia, and apoptotic and hypoxic cells were detected with appropriate markers followed by cell counts and group comparisons.

**Results:**

Treatment with ectoine resulted in RGC protection (*p* < 0.05) and reduced rate of apoptosis (*p* < 0.001) in hypoxia-treated retina organ cultures. However, the macroglia area and the amount of hypoxic, HIF-1α^+^ cells were unaffected by the ectoine treatment (*p* = 0.99). Treatment with hydroxyectoine also protected RGCs (*p* < 0.01) by inhibiting apoptosis (*p* < 0.001). In addition, the number of hypoxic, HIF-1α^+^ cells could be significantly reduced by treatment with hydroxyectoine (*p* < 0.05). The macroglia area on the other hand was unchanged after CoCl_2_ and treatment with hydroxyectoine.

**Conclusion:**

Both extremolytes had a protective effect on CoCl_2_-induced hypoxia in the porcine retina organ culture. Regarding the reduction of hypoxic stress, hydroxyectoine appears to be more effective. Thus, both extremolytes represent an interesting potential new therapeutic approach for patients with ocular diseases in which hypoxic processes play a significant role.

## Introduction

Hypoxia refers to a mismatch between oxygen demand and oxygen supply, so that a condition exists in which the tissue is not sufficiently supplied with oxygen. Both the structural and functional integrity of the retina depend on its oxygenation. As one of the most metabolically active tissues, oxygen is more rapidly consumed by the retina than by other tissues [1]. This makes the retina highly susceptible to hypoxic stress [2]. In many eye diseases, such as ischemic central retinal vein thrombosis and central retinal artery occlusion, hypoxia appears [3]. Furthermore, hypoxia is also involved in the development of diabetic retinopathy [4, 5] as well as glaucoma [6–8]. Glaucoma is the second leading cause of blindness worldwide [9]. The estimated number of people affected in 2010 was approximately 60.5 million [10]. By 2040, this number is expected to increase to around 111.8 million [11]. At the forefront of the disease is a progressive optic neuropathy with changes at the optic nerve head, gradual retinal ganglion cell (RGC) death, and visual field loss [9]. The cause of this degeneration is still unclear. An elevated intraocular pressure is still considered as the main risk factor [9], but there are increasing evidences that other pathological factors are involved. Another factor possibly involved in glaucoma pathomechanism is hypoxia [12, 13]. The upregulation of the transcription factor hypoxia-inducible factor-1 (HIF-1), in particular the stabilization of its oxygen-sensitive subunit HIF-1α, is a hallmark of hypoxic processes [14]. In most cell types without hypoxia, HIF-1α is undetectable due to the immediate degradation by the ubiquitin-proteasome pathway [15]. In contrast, hypoxic processes lead to an increase amount of HIF-1α, which enters the nucleus and in turn induces the expression of various hypoxic genes [16, 17]. Interestingly, in retinas of a high-pressure experimental rat glaucoma model, an upregulation of HIF-1α and some HIF-1α target genes was detectable [18]. Moreover, in glaucoma donor eyes, an increased expression of HIF-1α could be identified in RGCs and the optic nerve axons [19]. All these data indicate that HIF-1α expression might be a very crucial stage in glaucoma development and therefore a successful target for the implementation of neuroprotective drugs.

Cobalt belongs to the trace elements and is indispensable for the human organism but needed in low concentration. As part of vitamin B_12_, cobalt influences mitotic processes and is essential for neuronal integrity [20]. However, in high concentrations, cobalt has cyto- and neurotoxic effects [21]. Cobalt stabilizes HIF-1α by binding the oxygen-dependent region and prevents its degradation. Thus, it induces increased gene transcription that would otherwise occur only under hypoxic conditions [21]. Due to its properties as a hypoxia imitator, cobalt chloride (CoCl_2_) is commonly used to induce neurodegeneration in various models including retinal ganglion cell lines or neuroretina explants [22–26]. Previously, we developed an effective and reproducible hypoxia damage model by applying CoCl_2_ to porcine retina organ cultures. It is characterized by a strong degeneration, which is evidenced by a loss of neuronal cells of the inner retinal layers especially of RGCs. Furthermore, a reduced number of microglia cells, an increase of oxidative stress markers, and increased apoptosis mechanisms could be identified in this model [26].

Extremolytes are small, low-molecular, osmotically active substances that are used by so-called extremophilic organisms. Extremophiles include bacteria that are exposed to extreme temperature, pressure, radiation, pH, or salinity conditions [27]. The extremolytes protect the extremophiles against elevated salt concentrations and allow survival at high water temperatures as well as extreme drought [28]. In addition, they have been shown to stabilize protein structure and prevent denaturation of protein and nucleic acid [29, 30]. The extremolyte ectoine was first discovered in the halophilic sulfur bacterium *Ectothiorhodospira halochrloris*, which was isolated from the soda lake in Wadi an-Natrun in Egypt [31]. Hydroxyectoine was detected several years later in the Gram-positive bacterium *Streptomyces parvulus* and differs only in one functional group, the hydroxyl group on the C5 atom [32]. In contrast to drugs, the extremolytes do not have any pharmacological, metabolic, or immunologic mode of action. They achieve their effects in a purely physical manner by enhancing the H-bonds in water which results in a stabilization of cell membrane surfaces [33]. Based on the described inflammation-reducing and membrane-stabilizing properties of the compatible solutes, many potential applications are described. Currently, dermatology is the main application area as there are indications of positive effects on UVA-induced skin aging. Reduced release of inflammatory factors, protection of Langerhans cells, and a decrease in the number of apoptotic keratinocytes by ectoine after UV treatment could be demonstrated [34, 35]. Moreover, there is also a positive benefit of ectoine application in the context of inflammatory skin diseases, such as neurodermatitis [36]. Further indications of ectoine containing medical devices with clinically proven efficacy are inhalation solutions, nasal sprays, or eye drops against allergic diseases such as allergic rhinitis [37–39], allergic conjunctivitis [37], dry mucous membranes in the mouth and throat area [40], and respiratory diseases, like bronchial asthma, chronic obstructive pulmonary disease [41–43], and acute rhinosinusitis [44].

The goal of our study was to investigate for the first time possible neuroprotective effects of the extremolytes ectoine and hydroxyectoine in a retinal degeneration organ culture model. Hence, both extremolytes were applied to porcine retinal explants damaged by hypoxia. Cellular stress markers, apoptotic conditions, and RGCs were analyzed. In addition, macroglia cells were assessed. We found that both, ectoine and hydroxyectoine, had a protective effect on CoCl_2_-induced hypoxia in the porcine retina organ culture. Regarding the reduction of hypoxic stress, hydroxyectoine appears to be more effective.

## Material and methods

### Preparation of retinal explants

**Fig. 1:**
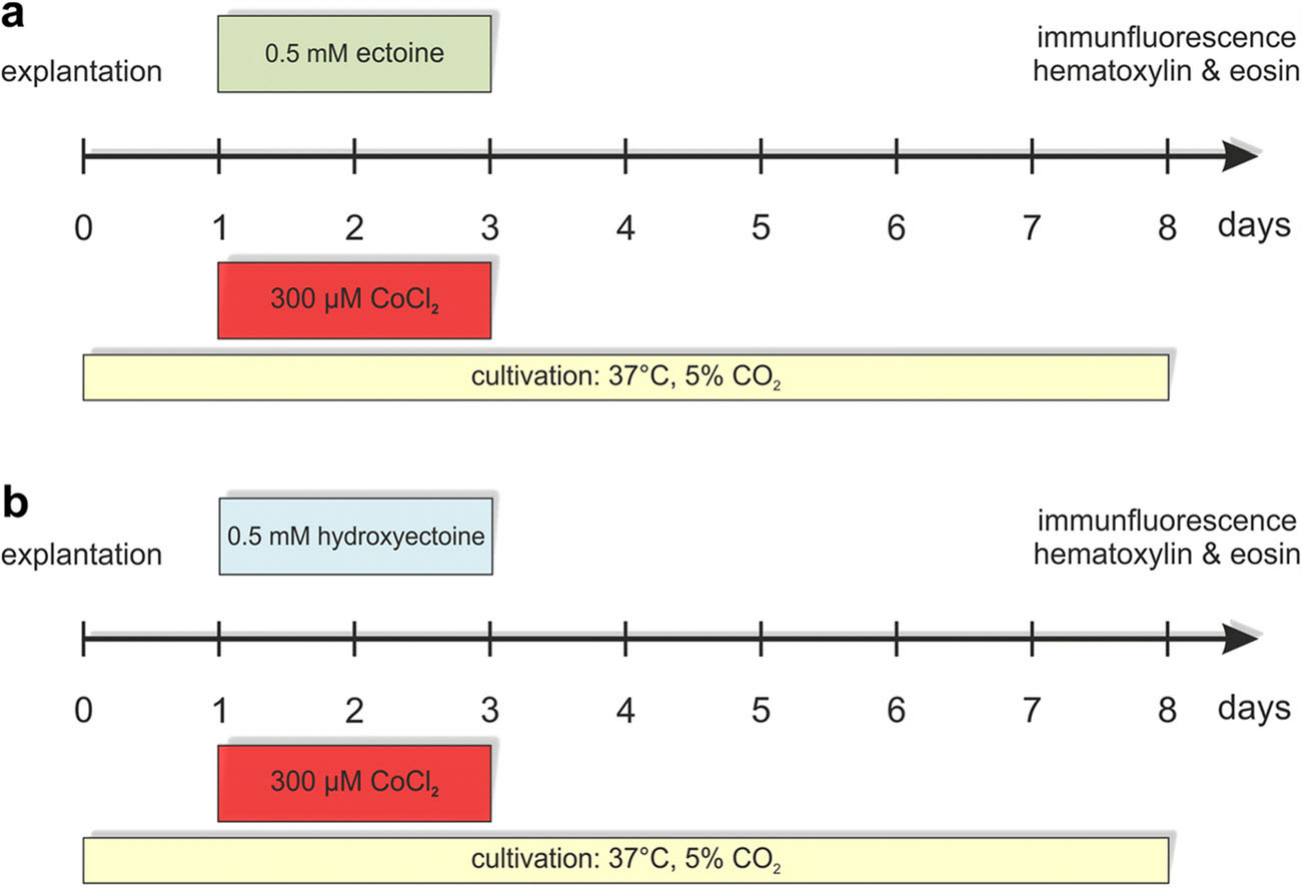
Experimental design. Cultivation of porcine retinas started with the explantation at day 0. At day 1, hypoxia was induced by adding the stressor CoCl_2_ (300 μM) to the medium of cultivated retinas in both studies. CoCl_2_ remained there for 48 h until day 3. At day 8, retinas were prepared for histological and immunohistochemical analyses. (**a**) To investigate possible neuroprotective effects of ectoine, 0.5 mM ectoine was added to retinas simultaneously to the stressor and remained for 48 h. Four groups were compared: control, ectoine, CoCl_2_, and ectoine + CoCl_2_. (**b**) Hydroxyectoine treatment was also added from day 1 to day 3 of cultivation, simultaneously to CoCl_2_. In this study, four groups were compared: control, hydroxyectoine, CoCl_2_, and CoCl_2_ + hydroxyectoine

The eyes of domestic pigs were obtained from the local abattoir (Bochum, Germany) and retinas were prepared within 3 h from enucleation. The preparation of retinal explants was performed as described previously [26, 45, 46]. Briefly, the eyeball was opened with scissors to separate the anterior parts of the eye from the eyecup. Subsequently, the eyecup was cut four times to obtain a cloverleaf-like shape. One retinal explant per leaf was punched out in the middle part of the quadrant using a dermal punch (*Ø* = 6 mm, Pfm medical AG, Cologne, Germany). Next, the explants were transferred to a Millicell culture insert (Millipore, Billerica, MA, USA) and cultured, with the photoreceptor layer facing the inserts, in Neurobasal-A medium supplemented with 0.8 mM l-glutamine, 2% B27®, 1% N-2 (all from Thermo Fisher Scientific, Schwerte, Germany), and 2% v/v penicillin/streptomycin (Sigma-Aldrich, St Louis, MO, USA) for 8 days in an incubator (37 °C, 5%CO_2_). The medium was exchanged completely at day 0, 1, 2, and 3. Additionally, half of the medium volume was replaced after 5 and 7 days. Starting from day 1, retina explants were incubated for 48 h with 300 μM CoCl_2_ (Sigma-Aldrich, St Louis, MO, USA) to induce a hypoxic environment [26, 47]. The treatment with the extremolytes was performed simultaneously to the CoCl_2_ stimulation and took 48 h in total (Fig. 1). In the first study, we applied ectoine (0.5 mM, Bitop AG, Witten, Germany; Fig. 1a). In a second experiment, hydroxyectoine (0.5 mM, Bitop AG, Witten, Germany; Fig. 1b) was used. The dose of 0.5 mM is based on preliminary data in which two different doses (0.5 mM and 10 mM) of ectoine and hydroxyectoine were tested for their uptake and their effects. On the one hand, it was shown that both ectoine doses were absorbed by the tissue. It was also observed that the higher dose of ectoine reduced the number of RGCs and increased apoptosis. The effect of hydroxyectoine was not quite as pronounced as that effect of ectoine but showed the same tendencies. Control groups for both studies were cultivated without the stressor CoCl_2_ and with or without additional ectoine/hydroxyectoine treatment. Four groups were compared in each study: control, CoCl_2_, control + ectoine/hydroxyectoine, and CoCl_2_ + ectoine/hydroxyectoine. At day 8, retinal explants were obtained for histological (*n* = 8–9/group) and immunohistochemical analyses (*n* = 8–9/group; Fig. 1).

### Histology

For histological evaluations, the retinal explants were treated for 15 min with 1.5% paraformaldehyde (Merck Millipore Ltd). Afterwards, incubation with 15% sucrose solution (VWR International GmbH, Ulm, Germany) for 15 min and 30% sucrose solution for 30 min was performed. Then, the explants were embedded in NEG-50 Tissue Tek medium (Fisher Scientific GmbH) and cryo-cross sections (10 mm thick) of the retina explants were cut on a microtome (Thermo Fisher, Waltham, MA, USA). The cryo-cross-sections were mounted on Histobond slides (Marienfeld, Lauda-Königshofen, Germany) and fixed in ice-cold acetone for 10 min.

The retinas were stained with hematoxylin and eosin (H&E) to visualize the different layers of the retina [48]. Two images in the central region of each retinal cross-section were taken in × 200 magnification (3 sections per sample, *n* = 6/group) using an Axio Imager.M1 microscope (Zeiss, Jena, Germany). Then, layers and the total retina were analyzed using the built-in measuring tool from Zen 2012 software (Zeiss). The thickness of the whole retina (excluding the outer segments), the ganglion cell layer (GCL), the inner plexiform layer (IPL), the inner nuclear layer (INL), outer plexiform layer (OPL), the outer nuclear layer (ONL), and the whole retina (excluding the outer segments) was measured in three regions per picture and these measurements were averaged.

### Immunohistology

For immunohistochemical analyses, retinal cross-sections (*n* = 8–9/group) were prepared as described under histology. Specific primary antibodies and matched secondary antibodies were used to identify different cell types and proteins of the retina (Table 1).

**Table 1:** Antibodies used for immunohistology analyses

Primary antibody			Secondary antibody		
Antibody	Company	Dilution	Antibody	Company	Dilution
Anti-Brn-3a	Santa Cruz	1:100	Donkey anti-goat Alexa Fluor 488	Dianova	1:500
Anti-cleaved caspase 3	Sigma-Aldrich	1:300	Donkey anti-rabbit Alexa Fluor 555	Jackson Immuno-Research	1:500
Anti-GFAP	Millipore	1:3000	Donkey anti-chicken Cy3	Millipore	1:500
Anti-HIF-1α	BD Bioscience	1:100	Donkey anti-mouse Alexa Fluor 555	Abcam	1:500
Anti-NeuN	Millipore	1:400	Donkey anti-chicken Alexa Fluor 488	Jackson Immuno-Research	1:500
Anti-Vimentin	Sigma-Aldrich	1:500	Goat anti-mouse Alexa Fluor 488	Invitrogen	1:500

First, the sections were rinsed in PBS (Santa Cruz Biotechnology, Inc., Dallas, TX, USA) before staining. For the Brn-3a and cleaved caspase-3 staining, the antigens were unmasked by boiling in citrate buffer (0.01 M, pH = 6; Bernd Kraft GmbH, Duisburg, Germany) for 10 min. Then, the sections were permeabilized and blocked with a mixture of serum samples (10–20% donkey or goat serum), 0.1–0.2% v/v Triton X-100 (Sigma-Aldrich Chemie GmbH), and PBS. Dilution of the primary antibodies (Table 1) was performed in the blocking solution and incubated at room temperature overnight. On the next day, the sections were incubated with Cy3/Alexa Fluor 555- or FITC-labeled secondary antibodies (Table 1) in the same mixture for 60 min. For all stainings, 4′,6 diamidino-2-phenylindole (DAPI; SERVA Electrophoresis GmbH, Heidelberg, Germany) was used to visualize the cell nuclei. Finally, the sections were mounted in Shandon™ EZ-Mount™ (Fisher Scientific GmbH). For each immunohistochemical staining, negative controls were performed with the secondary antibody only.

### Immunohistochemical examination

For all immunohistochemical stainings, six sections of each retina were stained. Four images (two central and two peripheral) were taken for each section with a fluorescence microscope (Axio Imager M1). In total, immunohistochemical analyses included 24 images per sample. Afterwards, these pictures were masked and cut with a predefined window (Corel PaintShop Pro X8, Corel, Ottawa, Ontario, Canada). For each retinal image, the number of cells labeled with Brn-3a, Brn-3a and cleaved caspase 3 colocalized cells, HIF-1α labeled cells in the whole retina as well as HIF-1α cells localized in the GCL, and NeuN and HIF1α colocalized cells in the GCL, were counted using ImageJ software (version 1.43u, NIH, Bethesda, MD, USA). Cell numbers of control retinas were set as 100% for all results regarding cell counting.

For GFAP and vimentin, the signal area was measured using an established protocol and an ImageJ software macro [49, 50]. Briefly, the masked pictures were converted to gray pictures and the background was subtracted (GFAP = ectoine study: 35.49 and hydroxyectoine study: 38.30; vimentin = ectoine study: 35.49 and hydroxyectoine study: 35.74). Then, lower and upper thresholds were determined (GFAP = ectoine: lower threshold: 8.98 and upper threshold: 100 and hydroxyectoine: lower threshold: 10.86 and upper threshold: 90.00; vimentin = ectoine: lower threshold: 3.77 and upper threshold: 100.00 and hydroxyectoine: lower threshold: 4.79 and upper threshold: 90.00). Using these thresholds, the signal area fraction per image was calculated with ImageJ. Figures of statistical analyses show the signal area fraction per image.

### Statistical analyses

Groups were compared by one-way ANOVA, followed by post hoc Tukey HSD test (Statistica V 13; Statsoft, Tulsa, OK, USA). Results are presented as scatter plots with means. A *p* value < 0.05 was considered as statistically significant. The level of significance was set to *p < 0.05, **p < 0.01, and ***p < 0.001.

## Results

### Ectoine

#### Increased survival of retinal ganglion cells through ectoine treatment

**Fig. 2:**
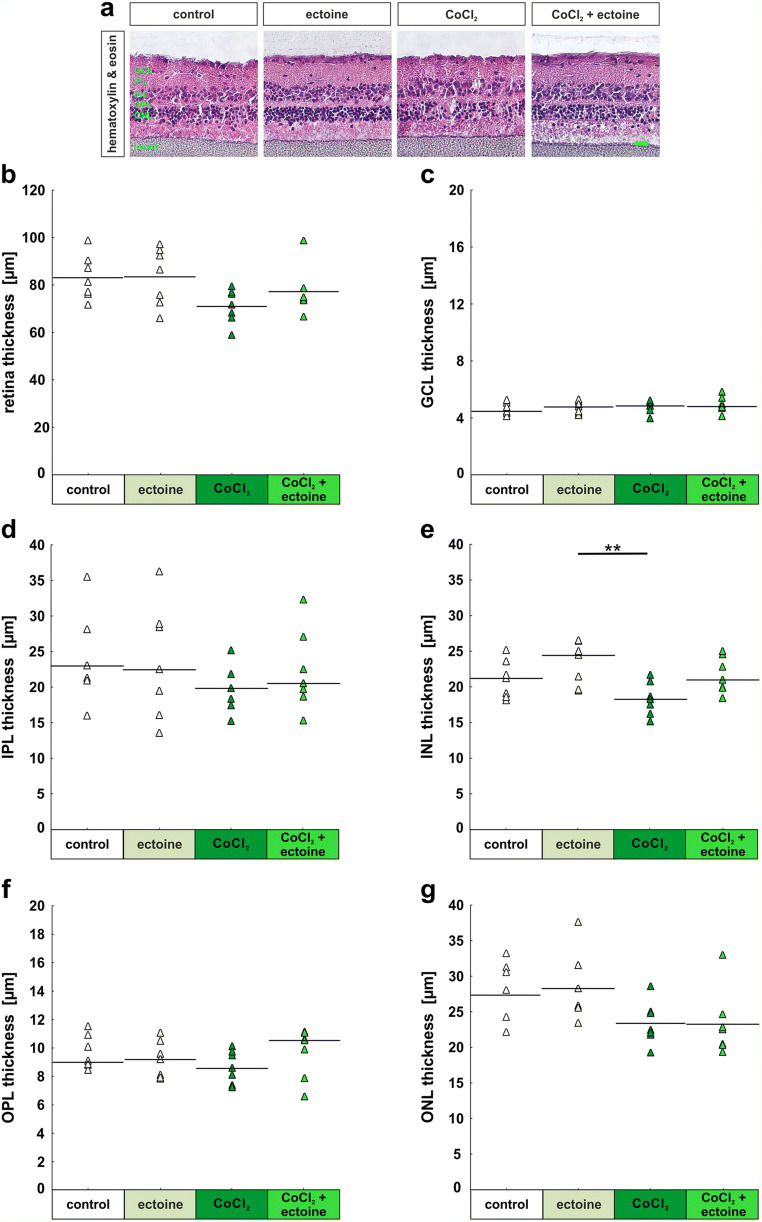
Comparable retinal structure. (**a**) To detect structural changes in the retina, cross-sections were stained with hematoxylin and eosin. Retinal layers were quite intact in all groups and revealed approximately the same number of cells. (**b**) The total retina thickness was comparable in the four groups. (**c**) The thickness of the GCL was also similar in all groups. (**d**) No significant differences were noted in IPL thickness. (**e**) The only significant difference in regard to the INL was noted between ectoine and CoCl_2_ samples. (**f**) The OPL thickness was rather the same in the evaluated groups. (**g**) In addition, the values of the ONL measurements were comparable in this study. GCL, ganglion cell layer; IPL, inner plexiform layer; INL, inner nuclear layer; OPL, outer plexiform layer; ONL, outer nuclear layer. Each triangle depicts an individual organ culture. The horizontal bar indicates the mean per group. Scale bar = 20 μm. **p < 0.01. *N* = 6/group

Hematoxylin and eosin staining can be used to investigate retinal structure. The organization of the retinal layers was not considerably altered through the cultivation or the substances, CoCl_2_ and ectoine, that were added (Fig. 2a). The thickness of the total retina in the control group was 83.1 ± 3.6 μm and 83.4 ± 4.6 μm in the ectoine group (*p* = 0.99; Fig. 2b). The retinal thickness of the CoCl_2_ (71.1 ± 2.7 μm; *p* = 0.13) and the CoCl_2_ + ectoine (77.2 ± 3.8 μm; *p* = 0.69) was also comparable with the control group. The GCL thickness was also similar in the ectoine (4.7 ± 0.4 μm; *p* = 0.92), the CoCl_2_ (4.7 ± 0.4 μm; *p* = 0.85), and the CoCl_2_ + ectoine group (4.9 ± 0.5 μm; *p* = 0.51), when compared with the control group (4.6 ± 0.5 μm; Fig. 2c). The IPL thickness in the ectoine (23.6 ± 3.0 μm; *p* = 1.00), the CoCl_2_ (19.6 ± 1.2 μm; *p* = 0.55), and the CoCl_2_ + ectoine (22.3 ± 2.2 μm; *p* = 0.95) was comparable with the control group (23.9 ± 0.5 μm; Fig. 2d). No significant differences were measured between the INL thickness of the ectoine group (23.3 ± 1.2 μm) and the control group (21.0 ± 1.0 μm; *p* = 0.4; Fig. 2e). The INL thickness of the CoCl_2_ (18.3 ± 0.9 μm; *p* = 0.25) and the CoCl_2_ + ectoine (21.6 ± 1.0 μm; *p* = 0.97) was also comparable with the control group. A significant difference was noted, when comparing the INL thickness of ectoine and CoCl_2_ samples (*p* = 0.009). OPL thickness in the ectoine (9.2 ± 0.49 μm; *p* = 0.90), the CoCl_2_ (8.6 ± 1.2 μm; *p* = 0.52), and the CoCl_2_ + ectoine (9.7 ± 0.7 μm; *p* = 1.00) was comparable with the control group (9.7 ± 0.5 μm; Fig. 2f). In regard to the ONL layer thickness, no significant differences were noted between any of the groups. Control samples had a mean ONL layer thickness of 27.3 ± 1.7 μm, ectoine samples of 28.3 ± 1.8 μm (*p* = 0.98), CoCl_2_ of 23.4 ± 1.1 μm (*p* = 0.34), and CoCl_2_ + ectoine of 23.3 ± 1.1 μm (*p* = 0.31; Fig. 2g).

**Fig. 3:**
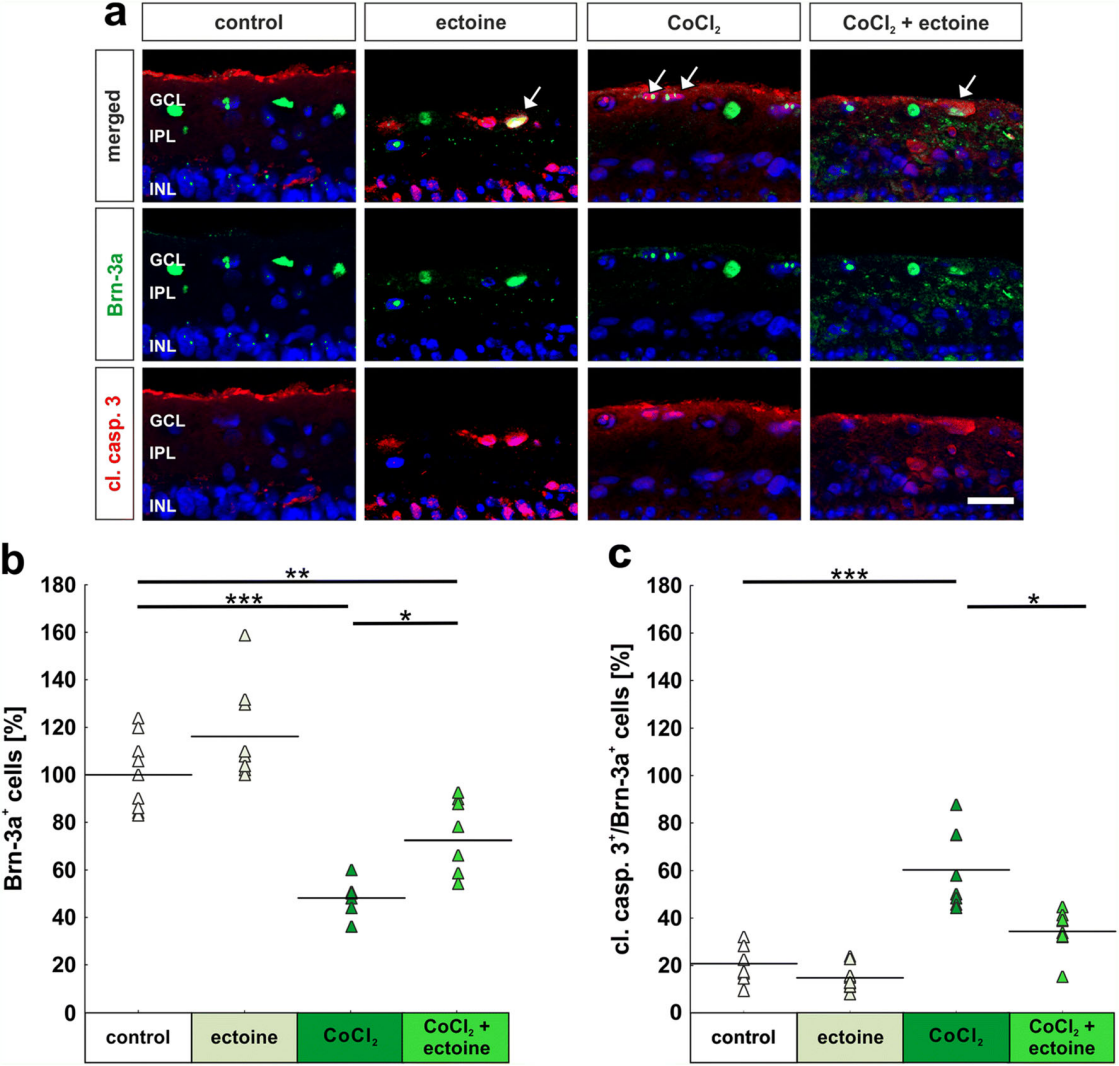
Rescue of retinal ganglion cells through ectoine. (**a**) RGCs were stained with a specific antibody against Brn-3a (green) and additionally with cleaved caspase 3 (cl. casp. 3; red, arrows label the signal) to detect apoptotic RGCs. Cell nuclei were visualized with DAPI (blue). (**b**) Cell counts of Brn-3a^+^ RGCs revealed a significant loss through CoCl_2_-induced hypoxia. Interestingly, ectoine treatment protected RGCs significantly. (**c**) RGCs loss in CoCl_2_-treated retinas was accompanied by an increased apoptosis rate, which was significantly inhibited through ectoine treatment. GCL, ganglion cell layer; IPL, inner plexiform layer; INL, inner nuclear layer. Each triangle depicts an individual organ culture. The horizontal bar indicates the mean per group. Cell counts are shown in percentage. Scale bars = 20 μm. *p < 0.05; **p < 0.01; ***p < 0.001. *N* = 8/group

To investigate the effects of ectoine on RGCs and their apoptosis rate, a double staining with anti-Brn-3a and anti-cleaved caspase 3-antibodies was performed (Fig. 3a). Cell counts of Brn-3a^+^ RGCs revealed no differences between the control group (100.0% ± 5.2% Brn-3a^+^ cells) and the ones treated with just ectoine (116.2% ± 6.6% Brn-3a^+^ cells; *p* = 0.15). In contrast, hypoxia induced a prominent loss of around 50% of RGCs in the CoCl_2_ group (48.2% ± 2.4% Brn-3a^+^ cells; *p* = 0.0002) even though the number of RGCs counted in the CoCl_2_ + ectoine group (72.47% ± 5.83% Brn-3a^+^ cells) was significantly lower than in the control group (*p* = 0.007). Moreover, ectoine led to a significant rescue of nearly 24% in comparison with the CoCl_2_ group (*p* = 0.02; Fig. 3b).

Results regarding apoptosis showed that the degenerative effect of CoCl_2_ is not limited to neurons of the ganglion cell layer, but occurred across all retinal layers. The results of the apoptosis rate of RGCs were consistent with those of the RGC counting (Fig. 3c). No differences were noted between the control (20.8% ± 2.7% cleaved caspase 3^+^ and Brn-3a^+^ cells) and the ectoine group (14.8% ± 1.7% cleaved caspase 3^+^ and Brn-3a^+^cells; *p* = 0.62). The addition of CoCl_2_ induced apoptotic processes in RGCs and led to a significant increase of nearly 40% of apoptotic RGCs (60.4% ± 5.9% cleaved caspase 3^+^ and Brn-3a^+^ cells; *p* = 0.0002). Interestingly, the treatment with ectoine clearly prevented apoptosis of some RGCs (34.4% ± 3.2% cleaved caspase 3^+^ and Brn-3a^+^ cells; *p* = 0.0003) in comparison with the CoCl_2_ group. Hardly, any differences were noted between the control and the CoCl_2_ + ectoine group regarding apoptotic RGCs (*p* = 0.06; Fig. 3c).

#### No effects of ectoine on the hypoxic stage of the retina

**Fig. 4:**
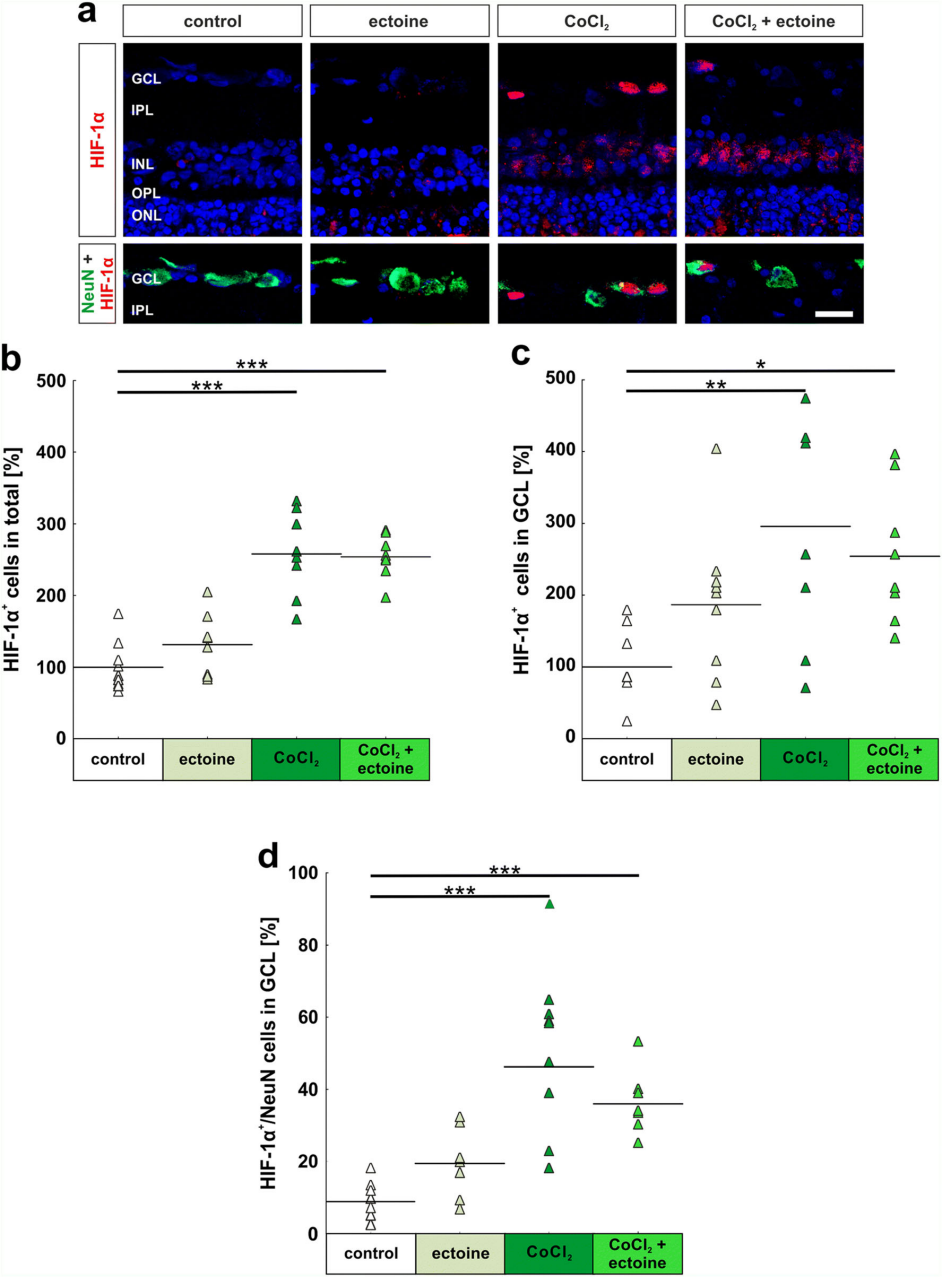
No effect of ectoine on number of hypoxic cells in retinas. (**a**) Neurons located in the GCL were stained with NeuN (green). Hypoxic cells were visualized by specific HIF-1α antibodies (red). Cell nuclei are shown in blue (DAPI). (**b**) CoCl_2_ led to an irreversible, significant increase of hypoxic cells in the total retina. Ectoine treatment did not have any inhibiting impact on the number of HIF-1α^+^ cells in all retinal layers. (**c**) Additionally, hypoxia significantly increased the number of HIF-1α^+^ cells in the GCL. Ectoine slightly decreased the number of hypoxic cells in the GCL. (**d**) Hypoxic neurons located in the GCL were evaluated by counting HIF-1α^+^ and NeuN^+^ cells. The addition of CoCl_2_ led to a significantly increased number of hypoxic neurons in the GCL. Ectoine had no impact on the number of hypoxic neurons. GCL, ganglion cell layer; IPL, inner plexiform layer; INL, inner nuclear layer; OPL, outer plexiform layer; ONL, outer nuclear layer. Each triangle depicts an individual organ culture. The horizontal bar indicates the mean per group. Cell counts are shown in percentage. Scale bar = 20 μm. *p < 0.05; **p < 0.01; ***p < 0.001. *N* = 8/group

A hallmark for oxidative stress is the stabilization of the transcription factor HIF-1α. To evaluate the hypoxic state of the retina, we performed immunohistochemical double staining using NeuN, a neuronal marker, for cells located in the GCL and HIF-1α as a marker for hypoxic cells (Fig. 4a). HIF-1α^+^ cells in all retinal layers (Fig. 4b), HIF-1α^+^ cells located in the GCL (Fig. 4c), and also HIF-1α^+^ and NeuN^+^ colocalized cells in the GCL (Fig. 4d) were evaluated.

Ectoine solely had no effect on the amount of HIF-1α^+^ cells in the total retina (control: 100.0% ± 11.6% HIF-1α^+^ cells; ectoine: 131.6% ± 13.6% HIF-1α^+^ cells; *p* = 0.40). In contrast, CoCl_2_ led in both stressed groups to a significantly increased number of HIF-1α^+^ cells in comparison with control retinas (CoCl_2_: 257.9% ± 20.8% HIF-1α^+^ cells; *p* = 0.0002; CoCl_2_ + ectoine: 253.9% ± 10.7% HIF-1α^+^ cells; *p* = 0.0002). Interestingly, ectoine had an effect on the hypoxic state of the cells, since no statistical differences were noted between the CoCl_2_ + ectoine and the CoCl_2_ group (*p* = 0.99; Fig. 4b). In regard to the number of HIF-1α^+^ cells located in the GCL, the ectoine group (186.2% ± 35.1% HIF-1α^+^ cells) had nearly twice high HIF-1α^+^ cell counts as the control group (100.0% ± 18.6% HIF-1α^+^ cells), but no statistical difference was seen between those two groups (*p* = 0.34). CoCl_2_-stressed retinas exhibited significantly more hypoxic HIF-1α^+^ cells in the GCL (CoCl_2_: 295.8% ± 55.1% HIF-1α^+^ cells; *p* = 0.005; CoCl_2_ + ectoine: 254.1% ± 33.6% HIF-1α^+^ cells; *p* = 0.036) than the control group. Even though retinas from the CoCl_2_ + ectoine group had slightly fewer HIF-1α^+^ cells than the solely CoCl_2_ group, no differences were seen comparing both groups (*p* = 0.86; Fig. 4c). Similar results as described before were seen when evaluating the number of hypoxic neurons, which were HIF-1α^+^ and NeuN^+^. Comparing the control (8.8% ± 1.7% HIF-1α^+^ and NeuN^+^ cells) and ectoine group (19.4% ± 2.8% HIF-1α^+^ and NeuN^+^ cells), no differences were noted (*p* = 0.18), whereas the addition of CoCl_2_ strongly increased the amount of hypoxic cells in the CoCl_2_ group (46.2% ± 6.4% HIF-1α^+^ and NeuN^+^ cells; *p* = 0.0002) as well as in the CoCl_2_ + ectoine group (35.9% ± 3.0% HIF-1α^+^ and NeuN^+^ cells; *p* = 0.0003). Again, ectoine had no lowering effect on the number of hypoxic neurons in comparison with the CoCl_2_ group (*p* = 0.25; Fig. 4d).

#### Absence of macroglial response in porcine retinas

**Fig. 5:**
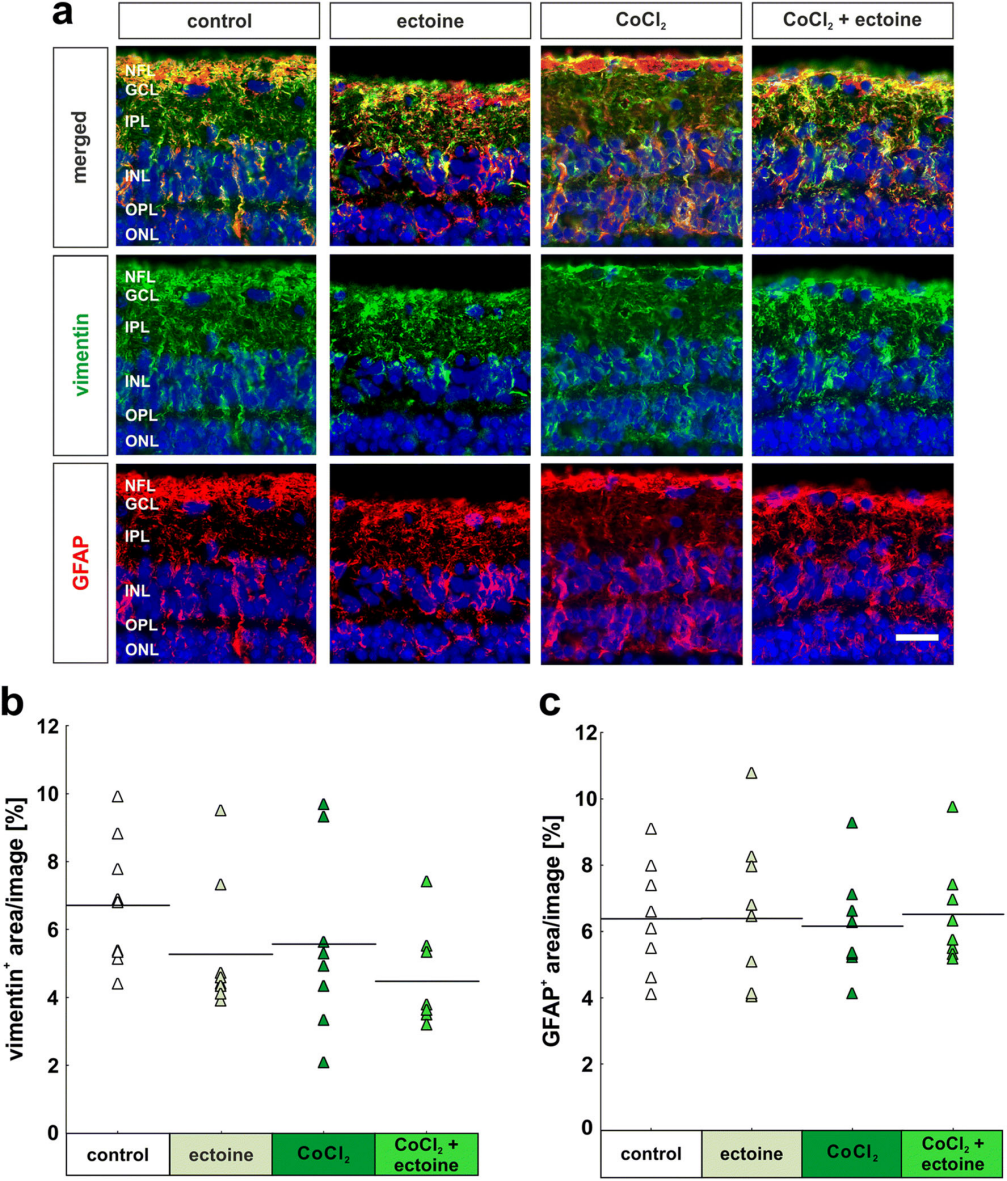
No macroglial response in retinas. (**a**) Müller glia were stained with a vimentin antibody (green), astrocytes with a GFAP antibody (red), and cell nuclei with DAPI (blue). Double staining of GFAP and vimentin is also displayed. (**b**) Evaluation of vimentin^+^ area revealed no differences within the four groups. (**c**) No alterations were seen comparing all groups in regard to GFAP^+^ area. NFL, nerve fiber layer; GCL, ganglion cell layer; IPL, inner plexiform layer; INL, inner nuclear layer; OPL, outer plexiform layer; ONL, outer nuclear layer. Each triangle depicts an individual organ culture. The horizontal bar indicates the mean per group. Scale bar = 20 μm. *N* = 8/group

Two important glial cell types of the retina are Müller cells and astrocytes. Müller cells span across the whole retina and are crucial for its health. They can be visualized using a vimentin antibody (Fig. 5a). Astrocytes, in contrast, are the main producer of glial fibrillary acidic protein (GFAP), a protein which is highly expressed in injured or diseased retinas. To investigate both cell types, retinal cross-sections were double stained using vimentin and GFAP antibodies. Exemplary images show the localization of both cell types, which are spanning across the retina. Interestingly, vimentin and GFAP appear to be mainly co-localized in the retina (Fig. 5a). Nevertheless, the immunoreactivities of both markers were analyzed individually.

The immunoreactivity of vimentin was comparable within all investigated groups (Fig. 5b). Neither solely ectoine (5.3% ± 0.6% vimentin^+^ area; *p* = 0.44), CoCl_2_ (5.6% ± 1.0%vimentin^+^ area; *p* = 0.67), nor CoCl_2_ + ectoine (4.5% ± 0.5% vimentin^+^ area; *p* = 0.14) had any effects on the vimentin^+^ area in these retinas compared with control ones (6.7% ± 0.6% vimentin^+^ area; Fig. 5b).

No differences regarding the GFAP^+^ area were seen within the groups (Fig. 5c). The immunoreactivity of GFAP in the control group (6.4% ± 0.2 GFAP^+^ area) was not altered in comparison with the ectoine group (6.4% ± 0.8% GFAP^+^ area; *p* = 1.00), the CoCl_2_ group (6.2% ± 0.6% GFAP^+^ area; *p* = 0.99), and the CoCl_2_ + ectoine group (6.5% ± 0.5% GFAP^+^ area; *p* = 1.00; Fig. 5c).

## Hydroxyectoine

### Retinal ganglion cell rescue and decreased apoptosis rate due to hydroxyectoine

**Fig. 6:**
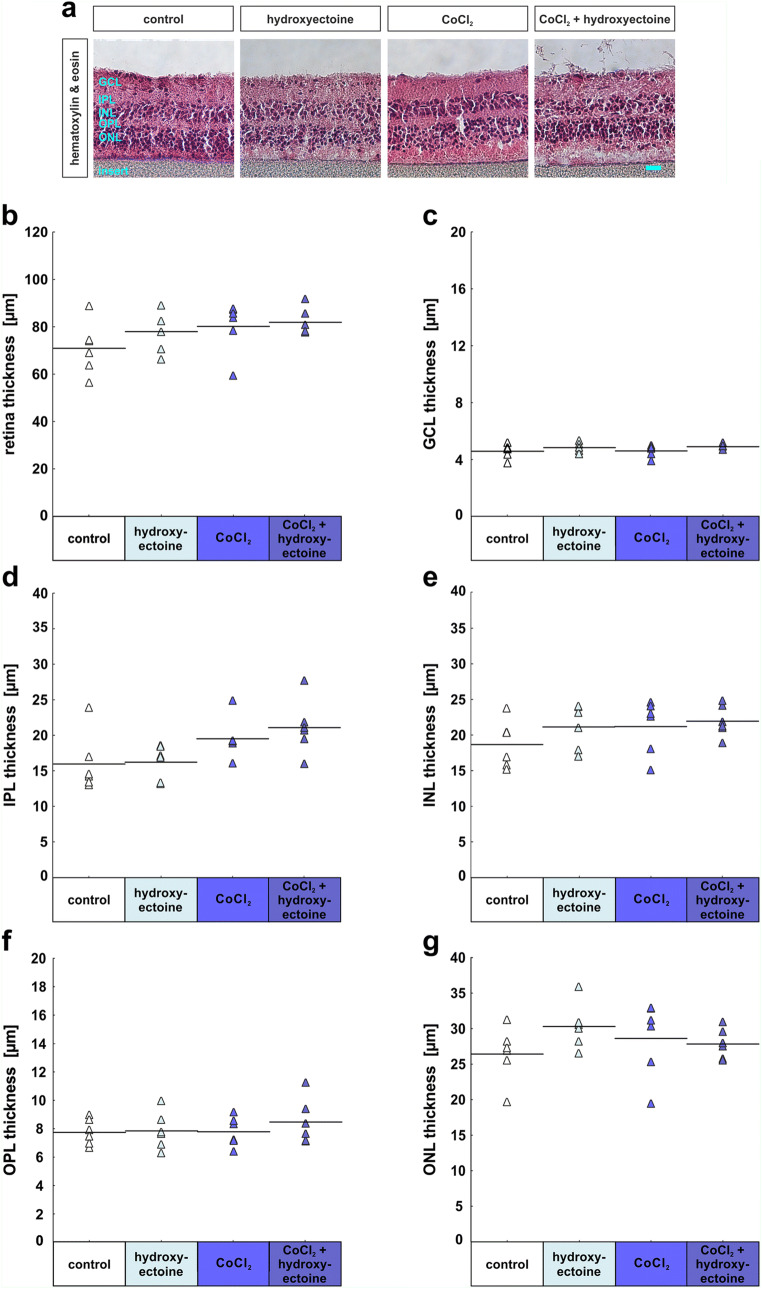
Retinal layers remain intact. (**a**) For an overview of retinal structure, hematoxylin and eosin staining was performed. Retinas of all groups remained intact during cultivation. (**b**) The total retinal thickness was similar in all groups. (**c**) No significant differences were noted in the GCL thickness between all analyzed groups. (**d**) The IPL thickness was also similar in all groups. (**e**) With regard to the ONL thickness, all groups have almost the same measured values. (**f**) The OPL thickness was rather the same in the evaluated groups. (**g**) In addition, the values of the ONL measurements were comparable in this study. GCL, ganglion cell layer; IPL, inner plexiform layer; INL, inner nuclear layer; OPL, outer plexiform layer; ONL, outer nuclear layer. Each triangle depicts an individual organ culture. The horizontal bar indicates the mean per group. Scale bar = 20 μm. *N* = 6/group

To analyze the possible protective effects of hydroxyectoine, the second extremolyte we tested, we again performed H&E staining to evaluate the integrity of the retina. In all four groups, the different retinal layers were well separated and identifiable, which indicated that the morphological integrity was given in all of the investigated retinas (Fig. 6a). In regard to the total retinal thickness, it was comparable in the control (70.9 ± 4.5), the hydroxyectoine (77.9 ± 3.4; *p* = 0.56), the CoCl_2_ (80.1 ± 4.4; *p* = 0.33), and the CoCl_2_ + hydroxyectoine group (81.8 ± 2.3; *p* = 0.20; Fig. 6b). The GCL thickness in the hydroxyectoine (4.8 ± 0.1; *p* = 0.64), the CoCl_2_ (4.6 ± 0.2; *p* = 1.0), and the CoCl_2_ + hydroxyectoine group (4.9 ± 0.1; *p* = 0.45) was not significantly different from control samples (4.6 ± 0.2; Fig. 6c). In regard to the IPL thickness, no significant differences were noted between any of the groups. Control samples had a mean IPL layer thickness of 16.1 ± 1.7 μm, hydroxyectoine samples of 16.3 ± 1.0 μm (*p* = 1.00), CoCl_2_ of 19.6 ± 1.2 μm (*p* = 0.29), and CoCl_2_ + hydroxyectoine of 21.2 ± 1.6 μm (*p* = 0.07; Fig. 6d). Moreover, no significant differences were measured between the INL thickness of the hydroxyectoine group (21.1 ± 1.3 μm) and the control group (18.7 ± 1.4 μm; *p* = 0.54; Fig. 2e). The INL thickness of the CoCl_2_ (21.2 ± 1.5 μm; *p* = 0.52) and the CoCl_2_ + hydroxyectoine (21.9 ± 0.9 μm; *p* = 0.30) were also comparable with the control group. In regard to the OPL layer thickness, no significant differences were noted between any of the groups. Control samples had a mean OPL layer thickness of 7.7 ± 10.4 μm, hydroxyectoine samples of 7.8 ± 0.5 μm (*p* = 1.0), CoCl_2_ of 7.8 ± 0.4 μm (*p* = 1.0), and CoCl_2_ + hydroxyectoine of 8.5 ± 0.7 μm (*p* = 0.74; Fig. 6f). The ONL thickness in the hydroxyectoine (30.2 ± 1.3 μm; *p* = 0.31), the CoCl_2_ (28.6 ± 2.2 μm; *p* = 0.75), and the CoCl_2_ + hydroxyectoine (27.8 ± 0.9 μm; *p* = 0.91) was again comparable with the control group (26.4 ± 1.6 μm; Fig. 6f).

**Fig. 7:**
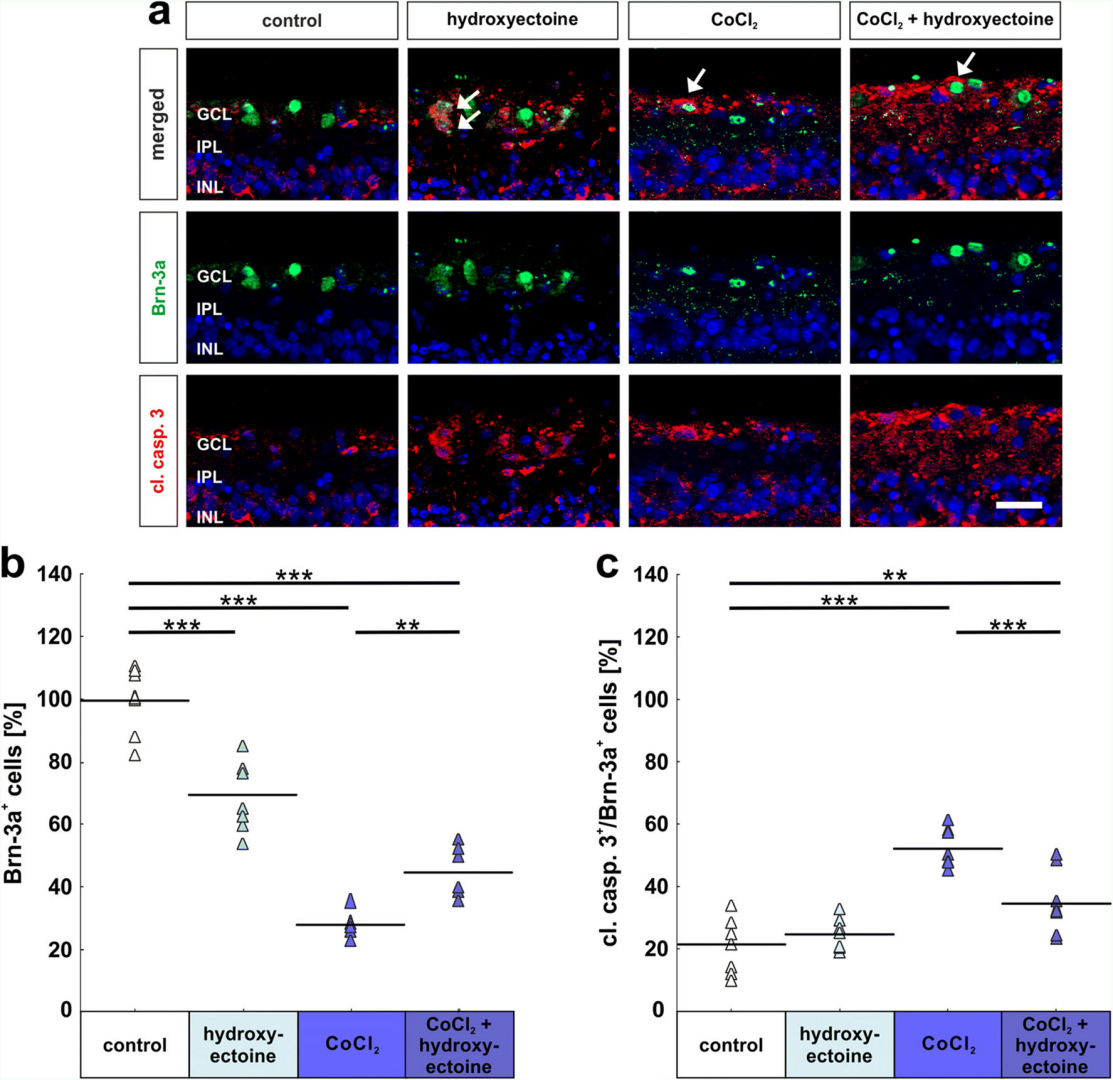
Hydroxyectoine protected retinal ganglion cells by inhibiting apoptosis. (**a**) Brn-3a antibodies were used to label RGCs (green). To visualize apoptotic RGCs, cleaved caspase 3 (cl. casp. 3, red, arrows label the signal) antibody was additionally applied. Cell nuclei were stained with DAPI (blue). (**b**) Again, CoCl_2_-induced hypoxia led to a significant loss of RGCs. Hydroxyectoine treatment strongly lowered this impact and led to a rescue of RGCs. (**c**) In accordance, RGC loss due to CoCl_2_ was accompanied by an increased apoptosis rate, which was significantly inhibited by hydroxyectoine. GCL, ganglion cell layer; IPL, inner plexiform layer; INL, inner nuclear layer. Each triangle depicts an individual organ culture. The horizontal bar indicates the mean per group. Cell counts are shown in percentage. Scale bars = 20 μm. **p < 0.01; ***p < 0.001. *N* = 8/group

RGCs and their apoptosis rate were analyzed immunohistochemically by specific antibodies Brn-3a and cleaved caspase 3 (Fig. 7a). Brn-3a^+^ cell numbers indicated a significant RGC loss just by adding hydroxyectoine to the medium (control: 100.0% ± 3.6% Brn-3a^+^ cells; hydroxyectoine: 69.7% ± 3.8% Brn-3a^+^ cells; *p* = 0.0002). The loss of RGCs due to CoCl_2_ was even more prominent (28.0% ± 1.3% Brn-3a^+^ cells; *p* = 0.0002), which was significantly lowered by hydroxyectoine (CoCl_2_ + hydroxyectoine: 44.9% ± 3.0% Brn-3a^+^ cells; *p* = 0.003). Even though a rescue effect by hydroxyectoine of around 16% was seen in CoCl_2_ + hydroxyectoine retinas in comparison with CoCl_2_ ones, retinas treated with CoCl_2_ + hydroxyectoine still exhibited significantly fewer RGCs than the control group (*p* = 0.0002; Fig. 7b).

Results regarding apoptosis showed that the degenerative effect of CoCl_2_ is not limited to neurons of the ganglion cell layer but occurs across all retinal layers. Despite the prominent loss of RGCs, no differences were seen in regard to the apoptosis rate when comparing the control group (21.4% ± 3.1% cleaved caspase 3^+^ and Brn-3a^+^ cells) and the hydroxyectoine group (24.6% ± 1.7% cleaved caspase 3^+^ and Brn-3a^+^ cells; *p* = 0.83). However, the loss of RGCs in CoCl_2_-treated retinas seemed to be provoked by apoptosis, since the apoptosis rate was twice as higher than in control ones (52.1% ± 2.1% cleaved caspase 3^+^ and Brn-3a^+^ cells; *p* = 0.0002). Hydroxyectoine treatment had a strong effect on the apoptosis rate and lowered it significantly in comparison with the CoCl_2_ group (34.5% ± 3.5% cleaved caspase 3^+^ and Brn-3a^+^; *p* = 0.0006), but it was still higher than in control retinas (*p* = 0.009; Fig. 7c).

### Reduced hypoxic processes in the retina after hydroxyectoine treatment

**Fig. 8:**
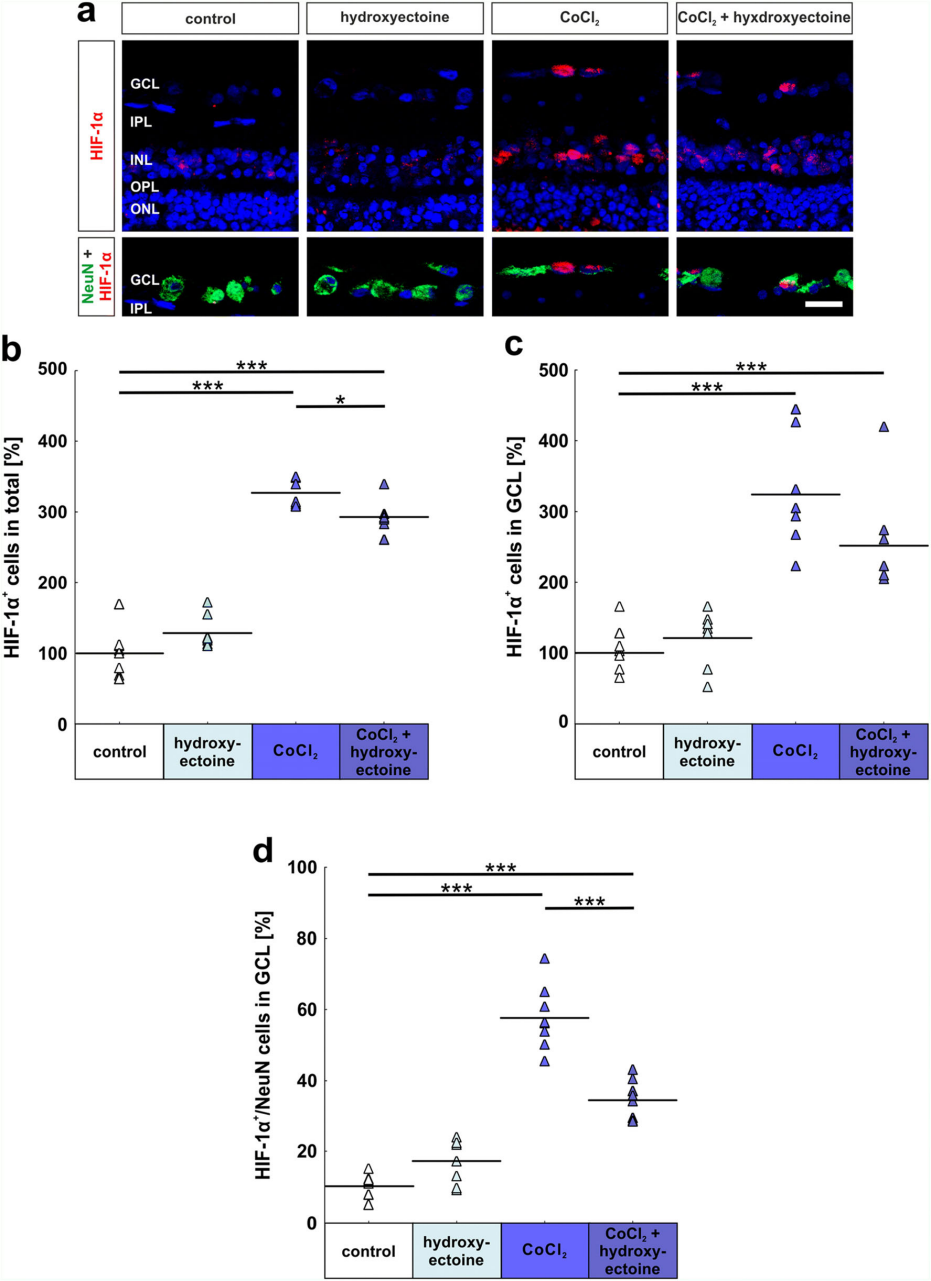
Reduction of hypoxia in retinas through hydroxyectoine. (**a**) Immunohistochemical staining of hypoxic neurons located in the retina was performed by NeuN (green) and HIF-1α (red) antibodies. Cell nuclei were visualized with DAPI and are shown in blue. (**b**) The amount of HIF-1α^+^ cells in the total retina was significantly increased due to the CoCl_2_-stressor. Hydroxyectoine led to a significantly decreased number of hypoxic cells in the retina. (**c**) Even if the effect of hydroxyectoine on hypoxic cells in the GCL was not as strong for the total retina, there was still a slight reduction of hypoxic cells after the treatment. (**d**) Also, the number of hypoxic neurons located in the GCL was strongly reduced through hydroxyectoine. GCL, ganglion cell layer; IPL, inner plexiform layer; INL, inner nuclear layer; OPL, outer plexiform layer; ONL, outer nuclear layer. Each triangle depicts an individual organ culture. The horizontal bar indicates the mean per group. Cell counts are shown in percentage. Scale bar = 20 μm. *p < 0.05; **p < 0.01; ***p < 0.001. *N* = 8/group

To investigate the effects of hydroxyectoine on hypoxia, retinal cross-sections were stained with specific NeuN and HIF-1α antibodies (Fig. 8a). The application of hydroxyectoine had no effect on the amount of HIF-1α^+^ cells in all retinal layers (control: 100.0% ± 11.7% HIF-1α^+^ cells; hydroxyectoine: 128.3% ± 7.8% HIF-1α^+^ cells; *p* = 0.12). In contrast, CoCl_2_ retinas without any additional treatment had three times more HIF-1α^+^ cells than control ones (326.6% ± 6.5% HIF-1α^+^ cells; *p* = 0.0002). Hydroxyectoine treatment lowered the stabilization of HIF-1α in retinal cells in comparison with the CoCl_2_ group (*p* = 0.048), but CoCl_2_ + hydroxyectoine-treated retinas still had more HIF-1a^+^ cells than control ones (292.8% ± 7.8% HIF-1α^+^ cells; *p* = 0.0002; Fig. 8b). Hypoxic cells located in the GCL showed similar results as the ones in the total retina, hydroxyectoine alone had no effects on HIF-1α (control: 100.0 ± 12.2 HIF-1α^+^ cells [%]; hydroxyectoine: 120.6% ± 13.4% HIF-1α^+^ cells; *p* = 0.89); CoCl_2_, irrespective of additional treatment, led to a significant increase in the number of HIF-1α^+^ cells (CoCl_2_: 323.8% ± 26.8% HIF-1α^+^ cells; *p* = 0.0002; CoCl_2_ + hydroxyectoine: 251.9% ± 25.6% HIF-1α^+^ cells; *p* = 0.0002). The statistical evaluation showed that hydroxyectoine had no positive effect on the amount of HIF-1α^+^ cells located in the GCL in comparison with CoCl_2_-treated retinas (*p* = 0.09; Fig. 8c). Regarding the number of hypoxic NeuN^+^ cells in the GCL, the effects of hydroxyectoine were more notable. Control and hydroxyectoine retinas displayed a similar number of HIF-1α^+^ and NeuN^+^ cells (control: 10.1% ± 1.2% HIF-1α^+^ and NeuN^+^ cells; hydroxyectoine: 17.2% ± 2.2% HIF-1α^+^ and NeuN^+^ cells; *p* = 0.13). Again, the effect of CoCl_2_ was very strong, leading to a significantly increased number of hypoxic neurons in the CoCl_2_ (57.5% ± 3.2% HIF-1α^+^ and NeuN^+^ cells; *p* = 0.0002) as well as in the CoCl_2_ + hydroxyectoine group (34.4% ± 2.0% HIF-1α^+^ and NeuN^+^ cells; p = 0.0002). Nevertheless, a protective effect of hydroxyectoine was observed, since hydroxyectoine and CoCl_2_-treated retinas had significantly fewer neurons in a hypoxic state than CoCl_2_ alone (*p* = 0.0002; Fig. 8d).

### No macroglial response

**Fig. 9:**
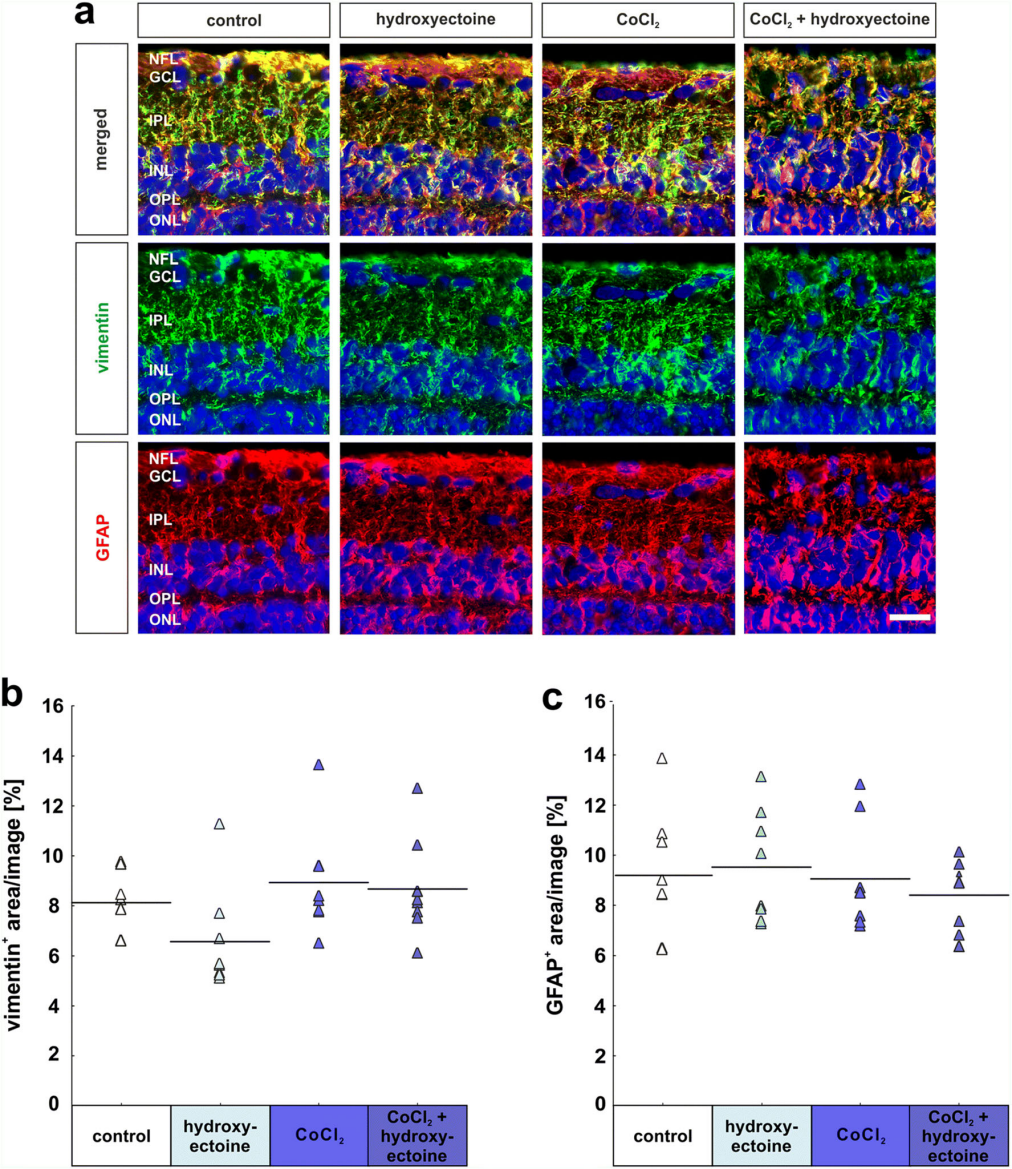
Macroglial response remained unaltered. (**a**) To visualize Müller glia and astrocytes, cross-sections were stained immunohistochemically using vimentin (green) and GFAP (red) individually and as a double staining. Cell nuclei are shown in blue (DAPI). (**b**) Analyses of vimentin^+^ area revealed no alterations within any of the groups. (**c**) Neither CoCl_2_ nor hydroxyectoine had any effects on GFAP signal area in retinas. NFL, nerve fiber layer; GCL, ganglion cell layer; IPL, inner plexiform layer; INL, inner nuclear layer; OPL, outer plexiform layer; ONL, outer nuclear layer. Each triangle depicts an individual organ culture. The horizontal bar indicates the mean per group. Scale bar = 20 μm. *N* = 8/group

Müller cells and astrocytes were investigated immunohistochemically by a double staining using vimentin and GFAP antibodies. Co-localization, namely vimentin and GFAP^+^ area, appears in yellow. Interestingly, a very high proportion of vimentin^+^ area is also GFAP^+^. Morphology and localization of labeled cells by these markers seem to be very similar. To investigate the macroglial response, vimentin and GFAP were analyzed individually (Fig. 9a). The vimentin^+^ area was not altered by any of the substances that were added to the medium. Neither hydroxyectoine solely (6.6% ± 0.7% vimentin^+^ area; *p* = 0.38) nor CoCl_2_ (8.9% ± 0.8% vimentin^+^ area; *p* = 0.83) or CoCl_2_ + hydroxyectoine (8.7% ± 0.7% vimentin^+^ area; *p* = 0.93) had any impact on Müller cells compared with controls (8.1% ± 0.4% vimentin^+^ area; Fig. 9b).

Also, the astrocytes expressing GFAP were not affected in any of the groups. The GFAP^+^ area was similar in the control (9.2% ± 0.9% GFAP^+^ area), the hydroxyectoine (9.5% ± 0.8% GFAP^+^ area; *p* = 0.99), the CoCl_2_ (9.1% ± 0.8% GFAP^+^ area; *p* = 1.0), and in the CoCl_2_ + hydroxyectoine group (8.4% ± 0.5% GFAP^+^ area; *p* = 0.88; Fig. 9c).

## Discussion

Extremolytes are defending substances that protect organisms from stress factors, such as heat, cold, high osmolarity, and dryness [27]. Each of the various members of the extremolyte family is preferably produced, absorbed, or accumulated from the environment under specific stress conditions [51]. The substances ectoine and hydroxyectoine, which are examined in more detail in this study, are mainly found under hypersaline conditions. Since they are substances compatible with the cellular metabolism that accumulate in the cytoplasm to balance external osmotic pressure, they are also called compatible solutes [52]. More and more studies are dedicated to the use of compatible solutes in the field of neurodegenerative diseases [53–56]. For example, in the context of Alzheimer research, ectoine and hydroxyectoine seem to be able to inhibit the aggregation of β-amyloid, which is responsible for plaque formation [56].

Although ectoine and hydroxyectoine are already part of current research in various areas, their effect on the retina and associated diseases was analyzed in this study for the first time. However, other osmotically active substances have been investigated for their potential therapeutic effect for retinal diseases. For example, a protective effect of betaine, which functions as a chaperone and protects against protein denaturation [57], has been demonstrated due to its inhibitory effect on the VEGF-mediated pathological neovascularization [58, 59].

Our first analyses of the osmolytes ectoine and hydroxyectoine showed a protective effect on the retina, evidenced by a significantly reduced loss of RGCs caused by hypoxia. Nevertheless, in comparison with control retinas, the loss of RGCs was not completely reversed. Interestingly, it has already been described in HaCaT cells that ectoine exerts its beneficial effects by activating the Akt-nuclear factor erythroid 2–related factor 2 (Nrf2) pathway, which in turn causes the expression of antioxidant proteins such as heme oxygenase 1 (HO-1) [60]. Moreover, it has been demonstrated before that the Nrf2/HO-1 system is one of the most important endogenous defense mechanisms against hypoxic stress [61] and also in the acute intraocular hypertension glaucoma model it was observed that a novel marine neuroprotectant agent can protect RGCs from acute ischemia/reperfusion injury by enhancing the Nrf2/HO-1 pathway [62]. Thus, it could also be possible that ectoine and hydroxyectoine protect the RGCs from CoCl_2_-induced hypoxia by activating the Akt/Nrf2 pathway.

Since macroglia plays an important role in many hypoxia-induced pathological mechanisms, the influence of ectoine or hydroxyectoine on this cell type was also investigated. Both substances had an influence neither on Müller cells nor on astrocytes. However, the sole administration of CoCl_2_ did not increase the macroglia area compared with the control group. One reason for the lack of this observation could be the separation from the optic nerve and the vascular supply, since astrocytes migrate into the retina via blood vessels and the optic nerve head [63]. Moreover, the cultivation time and the process of cultivation could be used as a declaration for the lack of macrogliosis. Previous studies on the one hand showed that CoCl_2_-induced degeneration of the porcine retina revealed a macrogliosis after 4 days, which disappeared over time [47]. On the other hand, investigations on porcine retina demonstrated that the cultivation alone triggered a macroglia response whether additional degenerative substance induced a reduction of the macroglia signals in comparison with cultured controls [26, 46, 64]. This implies that the process of cultivation seems to have the strongest effect on the macroglia.

To further elucidate the cellular factors which were involved in the observed effects of ectoine and hydroxyectoine, we performed studies on apoptosis via caspase 3 and on hypoxia via HIF-1α, which acts as a major player in the CoCl_2_-induced hypoxia cascade. A reduction in the apoptosis rate was detectable after ectoine and hydroxyectoine application. Interestingly, ectoine was able to reduce the apoptosis rate in such amount that a significant difference to the control retinas was no longer detectable. In contrast, the addition of hydroxyectoine reduced the rate of apoptosis, but a significant difference compared with the control group was still observable. These observations are in line with previous studies on the Machado–Joseph disease, which investigated the protective effects of the compatible solutes ectoine, hydroxyectoine, and betaine on apoptotic cell death produced by the truncated Machado–Joseph disease gene. Again, only ectoine, but not hydroxyectoine or betaine, decreased apoptotic features [54]. Furthermore, CoCl_2_ provoked a significant increase in HIF-1α^+^ cells. Interestingly, treatment with ectoine did not alter the number of hypoxic cells, neither in undamaged nor in CoCl_2_-degenerated retinas. The fact that ectoine was unable to reduce the stabilization of HIF-1α, but significantly reduced the number of apoptotic RGCs, suggests that ectoine is affecting the final part of the hypoxia-related pathway. This assumption is supported by the fact that numerous studies have previously shown that ectoine has protective effects on heat, cold, and dryness by stabilizing proteins and cell membranes [65–68]. In contrast to ectoine, a significant decrease in HIF-1α^+^ RGCs was noted after the administration of hydroxyectoine. These observations indicate that the additional hydroxyl group of hydroxyectoine causes further interactions associated with the stabilization of HIF-1 α, thereby affecting CoCl_2_-induced hypoxic retinal damage. Inter alia, a direct interaction between the additional hydroxy group and the hydrophilic head of the phospholipids is described, which disrupts processes at the cell membrane, such as the entrapment of vesicles [67]. Functional differences between ectoine and hydroxyectoine were not only detectable in our studies but also by other groups. In most cases, hydroxyectoine was superior to ectoine. For example, the application of hydroxyectoine caused a significant protein protection under heat stress in the enzyme lactate dehydrogenase model, while this effect was absent after ectoine treatment [69]. Moreover, *E. coli* K12 exhibited a higher survival rate after freeze-drying in the presence of hydroxyectoine than in the presence of ectoine [70]. Interestingly, examinations of the DNA melting temperature in the presence of ectoine or hydroxyectoine revealed that ectoine led to a lowering of the melting temperature, whereas hydroxyectoine increased it [71]. Since ectoine exerts a stronger influence on the apoptosis rate and hydroxyectoine on the number of hypoxic cells, the combination of both extremolytes might represent an approach that deserves further consideration. The combination of both extremolytes could increase the effects of the individual substances and thus the therapeutic effect. The positive effect of a combination therapy has already been demonstrated in the microbial growth of Streptomyces coelicolor A3(2). A mixture of 0.5 mM ectoine and 0.5 mM hydroxyectoine mediated a better osmoprotection than 1 mM ectoine or 1 mM hydroxyectoine alone [72]. Moreover, skin care products also use blends of ectoine and hydroxyectoine to combine the benefits of both extremolytes for the stability of phospholipid bilayer membranes [73].

In summary, in the present study, we were able to show for the first time that ectoine and hydroxyectoine are neuroprotective in a hypoxia-induced model of degenerative retinal ganglion cell damage. Thus, the application of both extremolytes appears to be an interesting new therapeutic approach for hypoxia-related eye diseases.
